# Dietary Oregano Oil Supplementation Improved Egg Quality by Altering Cecal Microbiota Function in Laying Hens

**DOI:** 10.3390/ani14223235

**Published:** 2024-11-12

**Authors:** Lili Xian, Yan Wang, Da Peng, Lei Zang, Yidan Xu, Yuanyuan Wu, Jingjing Li, Jing Feng

**Affiliations:** 1 Institute of Animal Husbandry and Veterinary Medicine, Xizang Academy of Agricultural and Animal Husbandry Science, Lhasa 850009, China; m17361552708@163.com (L.X.); wangyanls@hotmail.com (Y.W.); 13658917784@163.com (D.P.); m18089999024_1@163.com (L.Z.); 2 Key Laboratory of Animal Genetics and Breeding on Tibetan Plateau, Ministry of Agriculture and Rural Affairs, Lhasa 850009, China; 3 School of Life Science and Engineering, Southwest University of Science and Technology, Mianyang 621010, China; 15881681831@163.com; 4 Weifang Academy of Agricultural Sciences, Weifang 261071, China; wuyy167@126.com

**Keywords:** oregano oil, egg quality, intestinal morphology, late-phase laying hen, microbiota

## Abstract

Improving egg quality is one of the main objectives of the poultry industry. The objective of this study was to investigate the effects of dietary oregano oil supplementation with different concentrations on the egg quality, intestinal morphology and cecal microbiome of late-phase laying hens. A total of 300 55-week-old Snowy white laying hens were randomly divided into five groups and fed a basal diet (control) or basal diets supplemented with oregano oil at 25, 50, 75 and 100 mg/kg (O25, O50, O75 and O100) over a 100-day period. Compared with the control group, eggshell thickness and eggshell weight were increased significantly in eggs when oregano oil was added (*p* < 0.05). The highest content of polyunsaturated fatty acids (PUFAs) and C18:2n6c was observed in yolks with 25 mg/kg supplement among 5 groups (*p* < 0.05). The highest average content of riboflavin in egg yolk and thiamine in egg white was observed in the O25 group (*p* < 0.05). When the supplemental levels of oregano oil were 25 mg/kg and 50 mg/kg, the selenium and phosphorus content in the egg was significantly increased (*p* < 0.05). The ratio of villus height to crypt depth (V/C) and villus height of the small intestine was significantly increased compared with the chickens fed the basal diet (*p* < 0.05). Compared with the control group, the abundance of Megamonas was increased in the O50 group (*p* < 0.05). The unclassified_o__Bacteroidales in the O25 group were significantly higher than those in the other four groups (*p* < 0.05). These differential microorganisms were significantly correlated with egg quality characteristics. Dietary supplementation of oregano oil can significantly improve egg quality via gut microbiota alteration in laying hens.

## Simple Summary

We investigated the effects of dietary oregano oil supplementation with different concentrations on the egg quality, intestinal morphology and cecal microbiome of late-phase laying hens. The results showed that dietary oregano oil supplementation could enhance eggshell thickness and increase the content of PUFA, thiamine, riboflavin, selenium and phosphorus in egg yolk. Meanwhile, oregano oil supplementation had a good effect on the intestinal morphology and structure of laying hens in the late laying period. The differential microorganisms were significantly correlated with egg quality characteristics. Oregano oil may enhance the amino acid content of eggs by increasing the abundance of Synergistota, which in turn enhances egg quality. These results will support the use of oregano oil as a new feed additive to improve egg quality. Overall, 25 mg/kg of dietary oregano oil had the best effects.

## 1. Introduction

Eggs provide a cheap and easily accessible source of dietary protein and occupy a prominent position in the global food market [1]. Consumers are becoming increasingly concerned about the quality and nutritional value of eggs. The proteins, vitamins, and minerals found in eggs provide comprehensive benefits to maintain human health [2]. Egg quality is determined based on genetics and environmental conditions. Madalena et al. found that the eggs produced by commercial hens are more rounded, and the shells and yolks are darker than those of the four local breeds in Portugal [3]. Vlčková et al. investigated that storage time, the age of the hens and their interactions significantly affected egg weight, Haugh unit score and albumen pH [4]. Zhang et al. revealed that adding different concentration of vegetable oil to the diet could effectively improve egg quality [5].

Currently, phytogenic additives are recommended as safe and environmentally friendly alternatives and are widely used in the feed industry. Oregano oil is extracted from oregano, a perennial medicinal herb. It has a wealth of pharmacological properties, including antioxidant, antibacterial and anticancer properties [6,7]. It is one of the feed additives approved by the Ministry of Agriculture of China, with the characteristics of safety and high efficiency [8]. Therefore, it has been widely used in humans, mice, fish and chicken [9,10,11,12]. In poultry production, the beneficial effects of oregano oil have also been explored. Oregano at 1.0% enhanced productive performance, fecundity and hatching ability, and positively influenced the oxidative stability of shell eggs during storage [13]. Swanny et al. showed that the addition of 150 ppm oregano oil to the diet improved the percentage of egg production, yolk color, shell thickness and shell color [14]. Fei et al. suggested that egg production performance, feed conversion ratio, fatty acid content and microbial composition of eggs from late-laying hens could be improved by supplementation with 200 mg/kg oregano oil and 20 mg/kg cinnamaldehyde [15].

Egg quality in late-laying hens deteriorates due to impaired gut function, immune imbalance and gut microbiota imbalance caused by high-intensity production [16,17]. At the same time, the gut is the largest compartment of the immune system, and is involved in the protection of the host by providing a strong defense against invasion from the external environment [18]. The cecum of chickens harbors a complex microbiome [19,20]. Although several studies showed that dietary oregano oil supplementation could exert favorable effects on gut health in broilers [15,16,17], it is unknown whether oregano oil could improve egg quality by altering intestinal microbiota composition in later-laying hens. Therefore, the aims of this study were to investigate the effects of dietary oregano oil supplementation with different concentrations on the egg quality, intestinal morphology and cecal microbiome of late-phase laying hens.

## 2. Materials and Methods

### 2.1. Animals and Design

The trial was conducted at the Tibetan chicken-breeding base of the Tibet Autonomous Region Academy of Agricultural and Animal Husbandry Sciences. A total of 300 55-week-old snowy white chickens with similar body weight and good health were randomly divided into five groups that were further allocated to six replicates. Each replicate consisted of 10 broilers that were housed in cages. The dietary groups were as follows: the control group (group C) was fed a basal diet, and the experimental groups (groups O25, O50, O75 and O100) were fed a basal diet supplemented with oregano oil of 25, 50, 75 and 100 mg/kg, respectively. The oregano oil was purchased from Beijing Fedi Feed Technology Co., Ltd., Beijing, China. According to the “Feeding Standard for Chickens” (NY/T 33-2004) [21], the composition and nutritional level of the experimental basal diet are shown in Table 1. The experiment used 16 h of light and 8 h of darkness, and the light began at 06:00. Feed and water were provided ad libitum during the experimental period. The same environmental and management standards were applied to all birds. For specific feeding specifications refer to “Technical Specifications for Breeding of Lhasa White Chicken (Breed Group)” (DB54/T 0036-2009) [22].

### 2.2. Sample Collection

After a 100-day trial period, five eggs per replicate were randomly collected for quality parameter measurement. In addition, six chickens per group were randomly selected and transported to the slaughterhouse for sample collection. The duodenum, jejunum and ileum were sampled for intestinal morphology (*n* = 6). Intestinal segments were gently flushed with ice-cold phosphate-buffered saline (PBS, pH  =  7.4) to remove intestinal contents and placed in 4% paraformaldehyde for fixation. The contents of the cecum were then rapidly frozen in liquid nitrogen and stored at −80 °C until 16S rRNA sequencing (*n* = 6).

### 2.3. Egg Quality Parameters

Excluding abnormal eggs and double-yolked eggs, the following egg quality parameters were evaluated. Egg weight, yolk weight, and eggshell weight were measured using an electronic balance. Yolk thickness, eggshell thickness and air chamber diameter were measured by vernier caliper. The length and short diameter of the eggs were also measured by vernier caliper. The egg shape index was calculated using the formula egg shape index = length diameter of egg/short diameter of egg. The Haugh units (HU) were obtained using an egg quality analyzer (EMT-7300II, Robotmation, Tokyo, Japan).

The hydrolytic amino acid profile in the egg was measured according to the China National Standard (GB 5009.124-2016) [24]. The different tastes of amino acids could be divided into delicious amino acids (including aspartic acid and glutamic acid), sweet amino acids (including serine, glycine, proline, and alanine), and bitter amino acids (including lysine, valine, isoleucine, leucine, phenylalanine, histidine, arginine, and tyrosine). The free fatty acids in the egg were measured based on the method described by the China National Standard (GB/T 5009.168-2016) [25]. Riboflavin and thiamine were measured using high performance liquid chromatography following the method in the China National Standard (GB 5009.85-2016) [26] and (GB 5009.84-2016) [27], respectively. Glucose, total soluble sugar, total cholesterol, and triglyceride contents were measured using commercially available kits (Nanjing Jiancheng Bioengineering Institute, Nanjing, China). Crude protein was measured using kjeldahl determination, as described by the China National Standard (GB 5009.5-2016) [28], and crude fat was measured using soxhlet extraction, as described by the China National Standard (GB 5009.5-2016). The moisture proportion was calculated by drying and weighing. The ash proportion was calculated by burning and weighing. The potassium, calcium, phosphorus, magnesium, sodium, iron, selenium and zinc concentrations were determined by inductively coupled plasma-mass spectrometry according to the China National Standard (GB 5009.268-2016) [29,30].

### 2.4. Morphology of Small Intestine

After fixation for 24 h, the intestinal samples were dehydrated using ethanol and cleared with xylene, and then the samples were embedded in paraffin. Cross sections were made at a thickness of 5 μm and stained with hematoxylin-eosin. Morphological examination was applied at 40× magnification through an optical microscope (Nikon Eclipse Ci-L, Tokyo, Japan). Villus height and crypt depth of five intact well-oriented villi per segment were measured for each intestinal cross section using Image-Pro Plus 6.0 (Media Cybemetics, Rockville, MD, USA). The villus height to crypt depth ratio was then calculated. The average of the values for each cross section was used for further analysis.

### 2.5. Sequencing and Analysis of 16S rRNA Gene

Microbial DNA was extracted from the cecal samples using the E.Z.N.A.^®^ DNA kits (Omega Bio-tek, Norcross, GA, USA) according to the manufacturer’s protocol. On a 1% agarose gel, the concentration and purity of DNA were detected. The barcoded fusion forward primer 338F (5′-ACTCCTACGGGAGGCAGCAG-3′) and the reverse primer 806R (5′-GGACTACHVGGGTWTCTAAT-3′) were used to amplify the V3 and V4 hyper variable region of the 16S rRNA gene. High-throughput sequencing was conducted on an Illumina MiSeq PE300 platform by Beyotime Biotechnology Company (Shanghai, China).

The reads of each sample were demultiplexed, quality controlled by FATSP (version 0.12.0) and merged using Flash software (version 1.2.7). The UPARSE algorithm was used to cluster sequences into operational taxonomic units (OTUs) at a 97% similarity level. The OTUs were annotated based on Silva and UNITE taxonomy databases. QIIME software (https://qiime2.org) was utilized to generate species abundance at the phylum and genus levels. The R (version 4.1.3) was used to perform general statistical analysis and visualize results. Alpha diversity was estimated using the ACE, Chao1, Shannon and Simpson indices. Principal Coordinates Analysis (PCoA) was conducted to assess the differences in beta diversity between groups. Linear discriminant analysis (LDA) effect size (LEfSe) analysis was performed with a *p*-value < 0.05 for the Kruskal–Wallis test to compare the relative abundance of different taxa between groups.

### 2.6. Statistical Analysis

Statistical analysis was performed with SPSS 299.0 software (Chicago, IL, USA). One-way analysis of variance (ANOVA) was performed with Duncan’s post hoc test when the homogeneity of variance is significant (*p* < 0.05). All the analysis results were considered as the indication of statistical significance at *p* < 0.05.

## 3. Results

### 3.1. Egg Quality Parameters

The effects of different levels of oregano oil on egg quality are presented in Table 2. A significant difference was observed for eggshell thickness, air chamber diameter and eggshell weight (*p* < 0.05). The largest average air chamber diameter is the egg with 50 mg/kg oregano oil supplement (*p* < 0.05). Eggshell thickness and eggshell weight were increased significantly in eggs when oregano oil was added (*p* < 0.05). However, egg weight, yolk weight, Haugh unit, egg white thickness and yolk thickness did not differ significantly between the control and treatment groups (*p* < 0.05).

### 3.2. Amino Acids Profile

Data on the effect of oregano oil supplementation on the amino acid profile of eggs are shown in Table 3. A lack of statistical significance was observed for delicious amino acids, sweet amino acids and bitter amino acids in egg white and yolk (*p* > 0.05). The total essential amino acid (EAA) content in egg yolk was significantly lower in the O25 group than that of the other four groups (*p* < 0.05). However, there was no significant effect of the diet treatment on total EAA and individual EAA in egg white.

### 3.3. Fatty Acid Content

The levels of fatty acids in egg yolk are presented in Table 4. A total of 15 fatty acids were detected in yolk. Although the O50 group showed the greatest reduction in C4:0 content (*p* < 0.05), there was no significant difference in total saturated fatty acids and monounsaturated fatty acids in yolk (*p* > 0.05). The highest content of polyunsaturated fatty acids (PUFAs) and C18:2n6c were observed in yolk in the O25 group (*p* < 0.05).

### 3.4. Chemical Properties

The results of the present study highlighted the effect of different levels of oregano oil on chemical properties eggs (Table 5). There were significant differences in thiamine content in egg white and riboflavin content in yolk among all groups (*p* < 0.05). The highest average content of riboflavin in egg yolk and thiamine in egg white were observed in the O25 group (*p* < 0.05). There were significant differences in total soluble sugar in eggs among all groups (*p* < 0.05). When the concentration was 50 mg/kg, the total soluble sugar content in egg white and yolk was the highest (*p* < 0.05).

### 3.5. Mineral Elements

The mineral elements of eggs from various oregano oil supplementations are shown in Table 6. In egg white, the results revealed that groups treated with oregano oil had significantly increased phosphorus content (*p* < 0.05). When the supplemental levels of oregano oil were 25 mg/kg and 50 mg/kg, the selenium content in egg yolk was significantly increased (*p* < 0.05), but when the supplemental level of oregano oil was 75 mg/kg or above, the selenium level in egg yolk would start to drop. Oregano oil supplementation had no effect on potassium, calcium, magnesium, sodium, iron and zinc content (*p* > 0.05).

### 3.6. Intestinal Morphology

**Figure 1:**
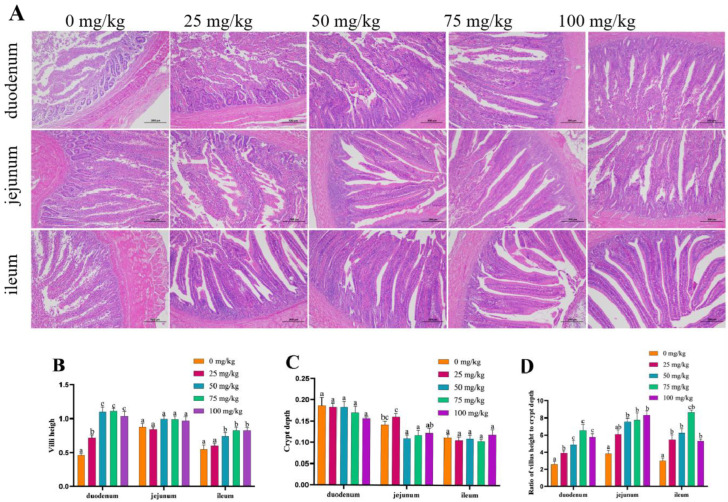
Small intestine morphology in broiler supplemented with oregano oil based on hematoxylin and eosin staining observed under 100× magnification: (**A**) intestinal morphological structure; (**B**) villus height V (μm); (**C**) crypt depth (μm); (**D**) ratio of villus height to crypt depth V/C. The data are presented as a mean and standard error of the mean. Bars with different superscript letter are statistically different (*p* < 0.05).

The morphological characteristics of intestinal tissues obtained from chickens in the different treatment groups are shown in Figure 1. All animals fed 0 mg/kg, 25 mg/kg, 50 mg/kg, 75 mg/kg and 100 mg/kg of oregano oil presented normal ranges for the heights and structures of villi and intestinal crypts. The villus height of the duodenum and ileum supplemented with oregano oil was significantly increased compared with the chickens fed the basal diet (*p* < 0.05) (Figure 1B). When oregano oil was added at concentrations of 50 mg/kg and 75 mg/kg, the crypt depth of the jejunum significantly decreased (*p* < 0.05). There was no significant effect of oregano oil on the crypt depth of the duodenum and ileum (*p* > 0.05) (Figure 1C). The ratio of villus height to crypt depth (V/C) of the duodenum, jejunum and ileum was significantly increased compared with the chickens fed the basal diet (*p* < 0.05) (Figure 1D). The results indicated that different levels of oregano oil supplementation in the basal diet had a good effect on the intestinal morphology and structure of snowy white chickens.

### 3.7. Cecum Microbial Diversity

**Figure 2:**
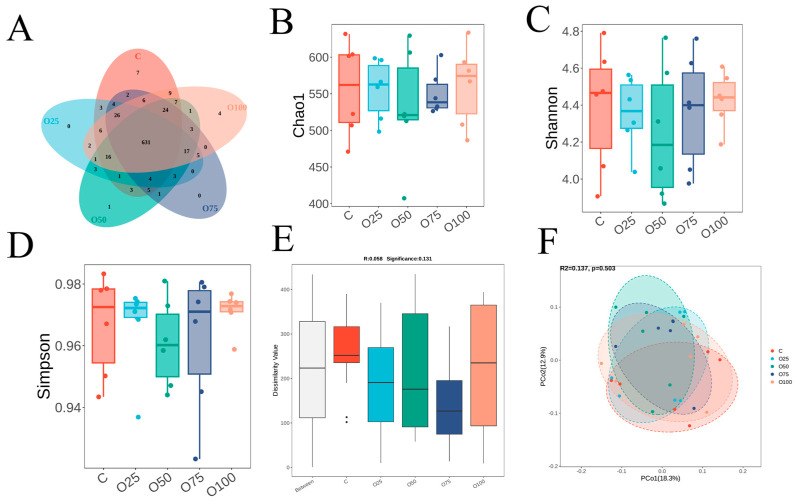
(**A**) Venn diagram illustrating the overlaps of microbial OTUs between the five groups. Alpha diversity of the cecum bacteria between the five groups: (**B**) Chao1 index; (**C**) Shannon index; and (**D**) Simpson index. (**E**) Differences between ANOSIM groups. (**F**) PCoA of taxonomical classifications of ruminal bacteria communities based on UniFrac distances.

A total of 644 OTUs were identified in all five cecum groups, whereas seven, zero, one, zero, and four specific operational taxonomic units (OTUs) were observed in the C, O25, O50, O75 and O100 groups, respectively (Figure 2A). The addition of oregano oil to the diet had no significant effect (*p* > 0.05) on the Chao1, Shannon, and Simpson indexes of bacterial richness and diversity (Figure 2B–D). ANOSIM analysis is a non-parametric test, based on the Bray–Curtis algorithm, which helps to determine whether differences between groups are observably greater than within group variations (Figure 2E). In this case, the R value can vary between −1 and 1, with values = 0.058 indicating significant between-group differences. The PCoA plot showed that there was no obvious separation among the five groups (Figure 2F).

**Figure 3:**
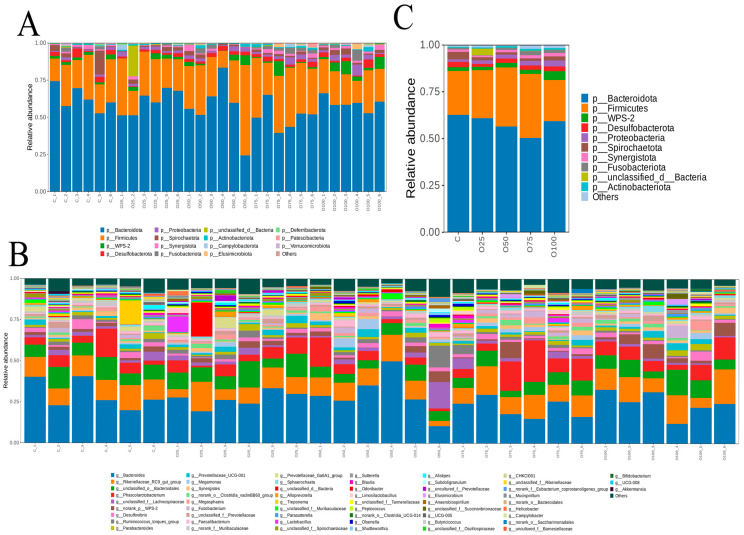
(**A**) Relative abundance of bacteria community proportions at the phylum. (**B**) Relative abundance of bacteria community proportions at the genus. (**C**) Histogram of the linear discriminant analysis (LDA) effect among the three groups, and the LDA score (log10) > 2 were shown.

At the phylum level, the cecal microbiota of the five groups was mainly composed of Bacteroidetes, Firmicutes, WPS-2, Desulfobacterota and Proteobacteria (Figure 3A). Among them, Firmicutes and Bacteroidetes occupied the largest proportion. There were no significant differences among all groups at the phylum level (*p* > 0.05). Analysis of the gut microbiota at the genus level showed that groups supplemented with 50 mg/kg oregano oil had an increased abundance of Megamonas compared with that in the control group (*p* < 0.05) (Figure 3B). The unclassified_o_Bacteroidales in the O25 group were significantly higher than those in the other four groups (*p* < 0.05). The abundance of Phascolarctobacterium was higher in the O75 group than that in the other four groups (*p* < 0.05). There was no difference in the abundance of Bacteroides and Rikenellaceae_RC9_gut_group (*p* > 0.05).

The linear discriminant analysis (LDA) effect size results showed that 21 genera were markers that distinguished the three groups of samples (Figure 3C). Five genera were greatly enriched in the C group: s_norank_g_UCG-008, g_UCG-008, s_norank_f_Rikenellaceae, g_norank_f_Rikenellaceae, and s_Alistipes_inops. Only two genera were enriched in the O25 group: s_norank_g_Paludicola and g_Paludicola. Three genera were significantly associated in the O50 group: s_norank_g_Sellimonas, s_norank_g_Solobacterium and g_Solobacterium. Three genera were enriched in the O75 group: s_norank_f_Tannerellaceae, g_norank_f_Tannerellaceae and s_Lactobacillus_ingluviei. Eight genera were significantly associated in the O100 group: s_norank_p_Proteobacteria, g_norank_p_Proteobacteria, f_norank_p_Proteobacteria, o_norank_p_Proteobacteria, c_norank_p_Proteobacteria, s_norank_g_Butyricimonas, g_Butyricimonas and f_Enterobacteriaceae.

### 3.8. Correlational Analysis Between Cecal Microbiota and Egg Quality

**Figure 4:**
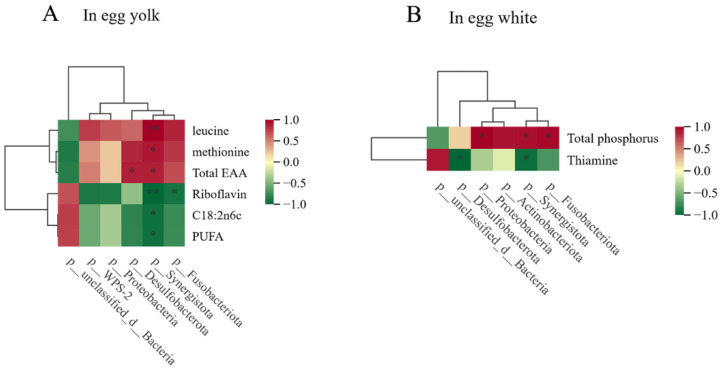
(**A**) Pearson’s correlation analysis between the cecal microbiota and egg quality parameters in yolk. (**B**) Pearson’s correlation analysis between the cecal microbiota and egg quality parameters in white. Red represents the positive correlation, and green represents the negative correlation. * *p* < 0.05. ** *p* < 0.01.

The correlations between cecal microbiota and egg quality parameters were analyzed (Figure 4). In egg yolk, correlation analysis revealed that the riboflavin, PUFA and C18:2n6c were negatively correlated with the abundance of Synergistota, whereas leucine, methionine and total EAA content were positively correlated with the abundance of Synergistota (*p* < 0.05). The findings suggest that oregano oil may enhance the amino acid content of eggs by increasing the abundance of Synergistota, which in turn enhances egg quality. Dietary Synergistota supplementation may be an effective strategy to improve egg quality in the poultry industry. Riboflavin was negatively correlated with Fusobacteriota (*p* < 0.05). Total EAA showed a positive correlation with Desulfobacterota (*p* < 0.05). In egg white, there was a negative correlation between thiamine and the abundance of Desulfobacterota and Synergistota (*p* < 0.05). The abundance of Fusobacteriota, Synergistota and Proteobacteria was positively correlated with total phosphorus (*p* < 0.05).

## 4. Discussion

Since oregano oil has been shown to have antioxidant, antibacterial and anticancer properties, it is widely used in the poultry industry [6,7,31]. In this study, the addition of oregano oil to the diet improved eggshell thickness in the late-laying period, which is consistent with previous reports [32,33]. Eggshell thickness, as an important index of egg quality, is important for egg transportation and storage [34]. Egg shell powder is considered a good source of highly bioactive calcium. This beneficial effect could be attributed to the active ingredients (i.e., thymol and carvacrol) in oregano oil, which improved intestinal health and nutrient utilization [33,35]. Amino acids play an important role in human nutrition and health. In this study, the effects of oregano oil on the amino acid composition of eggs were explored. Methionine and leucine are essential amino acids for protein synthesis in an animal body [36,37]. The results showed that a 25 mg/kg supplement of oregano oil reduced the content of methionine and leucine in the yolk. There was no significant effect of the other concentrations on essential amino acids.

Due to the close relationship between dietary lipids and the occurrence of diseases, the lipid composition of eggs has received much attention [38]. Previous studies presented the health benefits of polyunsaturated fatty acids (PUFA) on cardiovascular disease, diabetes, cancer, Alzheimer’s disease, dementia, depression and visual and neurological development [38,39,40]. In the present study, the PUFA content of group O25 and group O50 were significantly higher than that of the other three groups, implying the beneficial value of these two groups. The supplements of oregano oil changed the fatty acid content in egg yolk, probably by affecting serum cholesterol and lipid metabolism in the liver of laying hens [41,42,43,44,45]. Thiamine and riboflavin are water-soluble B-complex vitamins found in a variety of animal and vegetable products. They are found abundantly in lean pork, legumes, and eggs [46]. Adequate intake of riboflavin and thiamine can help preterm infants achieve adequate plasma concentrations and normal functional indices [47,48]. Riboflavin requirements appear to increase with exercise, dieting and dieting plus exercise in both young and older women engaged in moderate activity [49]. In this study, the highest average content of riboflavin was observed in the O25 group (*p* < 0.05). Therefore, consumption of eggs supplemented with 25 mg/kg oregano oil may have a positive effect on premature infants, pregnant women, kidney stone patients or athletes.

Phosphorus is a mineral that naturally occurs in many foods and is also available as a supplement. Phosphorus regulates the normal function of nerves and muscles [50]. The egg yolk contains 1% minerals, with phosphorus as the most abundant mineral component [51]. In our study, groups treated with oregano oil had significantly increased phosphorus content (*p* < 0.05). The essential trace mineral, selenium, is of fundamental importance to human health [52]. Se deficiency in humans increased the risk of some types of cancer and led to impaired mineralization of bones and teeth [53]. Humans generally increase their intake of Se by eating more Se-rich foods. Additionally, chicken eggs contain a large amount of selenium, and are an important source of selenium in the human diet [54]. In this study, the selenium content in yolk in group O25 and O50 was significantly increased (*p* < 0.05). In other words, consumption of eggs supplemented with 25 mg/kg and 50 mg/kg oregano oil may be helpful for people who are deficient in selenium and phosphorus.

Intestinal mucosal histology can be used to evaluate intestinal health [55]. Histological examination of the small intestine showed that the villus height and villus height/villus depth ratio were significantly increased when oregano oil was added. A previous study also suggested that oregano oil could increase the villus height and decrease the crypt depth, promoting the absorption of nutrients in the intestine [47]. At the same time, dietary supplementation of oregano oil and cinnamaldehyde could improve the performance of later-period laying hens, increase the villi absorption area, facilitate calcium absorption and increase eggshell thickness [33,48]. Thus, the oregano oil supplements probably increased thickness of the eggshell by improving intestinal morphology.

There are a large number of complex microbial flora in the intestinal tract of poultry, which can stimulate the growth and development of the gut, activate the immune system and promote the digestion and absorption of nutrients [56]. In the present study, there was no significant difference in the α-diversity among five groups. However, Feng et al. suggested that the microbiome composition was changed after oregano oil supplements [33]. Gao et al. also revealed that the combination of oregano oil and cinnamaldehyde increased the abundance of cecal flora and cecal microbial community structure [15]. The reason for this difference may be due to the fact that the concentration of oregano oil in this study is insufficient, and the combined effect of oregano oil and cinnamaldehyde is more obvious. The microbiota composition and specific taxa variation after the addition of oregano oil were then further analyzed. At the phylum level, the cecal microbiota was mainly composed of Bacteroidetes, Firmicutes, WPS-2, Desulfobacterota and Proteobacteria. Bacteroidetes are considered the leading factors for a healthy state, intestinal immunity, digestion and sophisticated homeostasis safeguarded by gut microbiota [57,58], while Desulfovibrio was considered to be harmful bacteria that inhibits the production of short-chain fatty acids [59,60]. In this study, Desulfobacterota was highly negatively correlated with thiamine and riboflavin in eggs. In addition, studies have shown that dietary C. butyricum supplementation could improve feed efficiency and yolk color [61]. Megamonas, hypermegale and Butyrivibrio species strains will improve immunity, thereby preventing and treating Behcet’s disease [62]. In this experiment, the content of Megamonas bacteria in the O50 group was significantly higher than that in other groups (*p* < 0.05), suggesting that supplementation of 50 mg/kg oregano oil may reduce the risk of Behcet’s disease.

## 5. Conclusions

In conclusion, the results of the present study indicate that dietary oregano oil supplementation could enhance eggshell thickness and increase the content of health-promoting PUFA, thiamine, riboflavin, selenium and phosphorus in egg yolk. Meanwhile, different levels of oregano oil supplementation in the basal diet had a good effect on the intestinal morphology and structure of laying hens in the late laying period. The dietary oregano oil supplementation regulated leucine, methionine, total EAA, riboflavin, C18:2n6c and PUFA content in egg yolk and thiamine and total phosphorus in egg white via cecal microbiota alteration. These results will support the use of oregano oil as a new feed additive to improve egg quality. Overall, dietary 25 mg/kg oregano oil had the best effects.

## Figures and Tables

**Table 1:** Comparison and chemical composition of the basal diet.

Ingredients (%)		Nutrient Levels ^2^	
Corn	62.70	ME (MJ/kg)	11.09
Soybean meal (CP 43%)	26.30	CP (%)	16.61
CaHPO4	1.00	CF (%)	3.31
DL-Methionine	0.10	Available phosphorous, % (%)	0.35
Limestone	8.50	Lysine (%)	0.85
Choline chloride (70%)	0.10	Methionine (%)	0.35
NaCl	0.30	Ca (%)	3.50
^1^ Premix	1.00	Na (%)	0.01
Total	100		

^1^ Premix, supplied each kg consists of: VA 10,000 IU, VD3 3000 IU, VE 20 IU, VK 32 mg, thiamine 1 mg, riboflavin 8 mg, calcium pantothenate 40 mg, niacin 32.5 mg, pyridoxine 8 mg, biotin 2 mg, folic acid 1.5 mg, VB12 5 mg, choline 500 mg, Mn 70 mg, I 1 mg, Fe 80 mg, Cu 8 mg, Zn 80 mg and Se 0.3 mg. ^2^ Nutrient levels, Calculated according to NRC (1994) [23].

**Table 2:** Effects of oregano oil on egg quality parameters.

Item	Concentration (mg/kg)					SEM	*p* Value
	0	25	50	75	100		
Egg weight (g)	52.02	51.21	54.03	52.97	52.40	0.51	0.53
Yolk weight (g)	17.23	16.39	18.05	17.53	16.77	0.22	0.14
Eggshell weight (g)	5.39 ^a^	6.38 ^b^	6.42 ^b^	6.38 ^b^	6.35 ^b^	0.12	0.01
Haugh unit	72.00	84.83	83.33	82.66	79.00	1.60	0.07
Egg white thickness (mm)	5.82	7.24	7.27	7.19	6.51	0.22	0.17
Yolk thickness (mm)	17.18	18.11	18.69	18.84	18.00	0.21	0.08
Eggshell thickness (mm)	0.25 ^a^	0.33 ^b^	0.33 ^b^	0.29 ^b^	0.34 ^b^	0.011	0.00
Air chamber diameter (mm)	16.01 ^a^	15.92 ^a^	17.21 ^b^	17.09 ^ab^	16.03 ^a^	0.215	0.04
^1^ Egg shape index	1.33	1.31	1.30	1.33	1.29	0.21	0.75

^a,b^ Data within the same row marked with different small letters above the data indicate statistically significant differences (*p* < 0.05), while the same or no small letters indicate non-significant differences (*p* > 0.05). ^1^ Egg shape index = Ratio of length diameter of egg to short diameter of egg.

**Table 3:** Effects of oregano oil on amino acid composition of egg.

Items (mg/g)	Concentration (mg/kg)					^1^ SEM	*p* Value ^2^
	0	25	50	75	100		
Egg white							
Glutamic acid	14.16	12.81	12.62	14.08	13.56	0.45	0.79
Aspartic acid	11.95	10.93	10.60	11.81	11.65	0.35	0.75
Glycine	5.68	5.42	5.15	5.42	5.60	0.23	0.97
Alanine	10.22	9.32	9.08	10.28	9.77	0.32	0.76
Serine	12.39	11.33	11.06	12.45	11.93	0.46	0.87
Proline	5.46	5.11	5.31	5.69	5.23	0.23	0.96
Leucine	0.21	0.15	0.16	0.18	0.19	0.01	0.23
Isoleucine	13.85	12.70	12.75	14.11	13.23	0.54	0.92
Valine	10.34	9.49	6.22	10.67	9.81	0.77	0.41
Phenylalanine	6.63	6.09	6.46	6.99	6.29	0.26	0.89
Tyrosine	3.55	3.37	3.50	3.78	3.41	0.15	0.94
Histidine	3.70	3.59	3.01	3.44	3.75	0.16	0.66
Lysine	7.90	7.19	6.83	7.97	7.58	0.32	0.83
Arginine	9.02	6.66	8.38	9.26	8.73	0.60	0.73
Methionine	3.39	2.92	3.28	3.80	3.17	0.17	0.61
Threonine	6.90	6.20	6.20	7.14	6.63	0.27	0.80
Cysteine	6.81	6.19	6.48	7.11	6.45	0.27	0.87
Total EAA	49.21	44.74	41.91	50.86	46.89	1.98	0.69
Delicious amino acids	26.11	23.74	23.22	25.89	25.21	0.80	0.77
Sweet amino acids	33.75	31.17	30.60	33.84	32.53	1.20	0.91
Bitter amino acids	55.20	49.23	47.32	56.39	52.99	2.33	0.76
Yolk							
Glutamic acid	14.00	13.62	13.14	13.39	13.91	0.14	0.23
Aspartic acid	11.18	10.87	10.35	10.51	10.91	0.12	0.13
Glycine	7.98	7.45	7.52	7.69	7.96	0.09	0.21
Alanine	9.80	9.58	9.00	9.00	9.65	0.14	0.17
Serine	17.02	16.42	16.38	16.82	17.47	0.17	0.20
Proline	9.59	9.44	9.67	10.07	9.62	0.11	0.50
Leucine	0.21 ^abc^	0.19 ^a^	0.20 ^ab^	0.22 ^bc^	0.23 ^c^	0.00	0.04
Isoleucine	16.95	16.41	16.34	16.63	16.87	0.11	0.29
Valine	12.04	11.85	11.83	11.91	12.01	0.06	0.85
Phenylalanine	7.23	7.13	7.23	7.39	7.19	0.07	0.84
Tyrosine	5.26	5.25	5.27	5.49	5.45	0.05	0.45
Histidine	3.60	3.46	3.30	3.40	3.73	0.08	0.49
Lysine	9.09	8.73	8.47	8.43	9.04	0.12	0.23
Arginine	9.87	9.77	9.81	9.74	9.87	0.07	0.98
Methionine	6.27 ^b^	1.93 ^a^	3.83 ^ab^	6.41 ^b^	6.22 ^b^	0.62	0.04
Threonine	9.41	9.38	9.27	9.50	9.78	0.07	0.25
Cysteine	9.40	9.08	9.09	9.34	9.39	0.06	0.29
Total EAA	61.21 ^b^	55.63 ^a^	57.72 ^ab^	60.49 ^b^	61.33 ^b^	0.78	0.04
Delicious amino acids ^3^	25.18	24.49	23.48	23.89	24.82	0.25	0.17
Sweet amino acids ^4^	44.39	42.89	42.57	43.57	44.70	0.34	0.19
Bitter amino acids ^5^	64.25	62.79	62.44	63.21	64.38	1.23	0.49

^a–c^ Data within the same row marked with different small letters above the data indicate statistically significant differences (*p* < 0.05), while the same or no small letters indicate non-significant differences (*p* > 0.05). ^1^ SEM: standard error means. ^2^ Overall treatment *p*-value. ^3^ Delicious amino acids = Aspartic acid + Glutamic acid. ^4^ Sweet amino acids = Serine + Glycine + Proline + Alanine. ^5^ Bitter amino acids = Lysine + Valine + Isoleucine + Leucine + Phenylalanine + Histidine + Arginine + Tyrosine.

**Table 4:** Effects of oregano oil on fatty acid composition of egg.

Items (mg/L)	Concentration (mg/kg)					SEM	*p* Value
	0	25	50	75	100		
Yolk							
C4:0	8.51 ^bc^	7.48 ^abc^	3.40 ^a^	5.19 ^ab^	10.58 ^c^	0.87	0.04
C6:0	8.75	8.02	5.83	5.52	9.47	0.57	0.07
C11:0	3.78	2.08	2.14	1.86	5.25	0.48	0.08
C14:0	4.71	19.59	15.65	3.69	1.16	2.83	0.13
C14:1	0.00	3.55	3.02	0.00	0.00	0.73	0.32
C16:0	314.59	1246.78	903.54	259.65	83.01	182.40	0.21
C16:1	42.78	168.30	132.43	30.93	12.11	26.51	0.25
C17:0	0.00	10.28	8.14	3.25	0.00	1.54	0.08
C18:0	86.24	375.21	263.48	87.74	25.84	52.70	0.17
C18:1n9c	239.24	1080.65	914.37	227.20	65.73	180.40	0.28
C18:2n6c	198.25 ^a^	1148.39 ^b^	875.38 ^b^	215.46 ^a^	51.08 ^a^	136.31	0.01
C20:2	0.00	8.54	6.70	2.17	0.00	1.35	0.12
C20:3n3	1.06	6.86	4.49	0.49	0.00	1.00	0.10
C20:4n6	15.90	63.97	63.61	18.36	7.31	11.57	0.36
C23:0	5.71	8.03	6.13	6.19	4.73	0.61	0.59
^1^ SFA	432.29	1677.46	1208.31	373.08	140.04	239.42	0.20
MUFA ^2^	282.01	1252.50	1049.82	258.13	77.84	207.54	0.28
PUFA ^3^	215.21 ^a^	1227.75 ^b^	950.18 ^b^	236.48 ^a^	58.38 ^a^	148.48	0.01

^a–c^ Data within the same row marked with different small letters above the data indicate statistically significant differences (*p* < 0.05), while the same or no small letters indicate non-significant differences (*p* > 0.05). ^1^ SFA: saturated fatty acid (SFA = C4:0 + C6:0 + C11:0 + C14:0 + C16:0 + C17:0 + C18:0 + C23:0). ^2^ MUFA: monounsaturated fatty acid (MUFA = C14:1 + C16:1 + C18:1n9c). ^3^ PUFA: polyunsaturated fatty acid (PUFA = C18:2n6c + C20:2 + C20:3n3 + C20:4n6).

**Table 5:** Effects of oregano oil on chemical properties contents of egg.

Items	Concentration (mg/kg)					SEM	*p* Value
	0	25	50	75	100		
Egg white							
Glucose (mg/g)	4.26	3.93	3.49	3.99	3.99	0.19	0.82
Crude protein (%)	12.40	11.79	13.02	13.02	11.85	0.34	0.70
Total soluble sugar (mg/g)	7.42 ^a^	7.71 ^a^	9.74 ^b^	7.67 ^a^	7.79 ^a^	0.26	0.00
Crude fat (%)	0.51	0.56	0.56	0.58	0.54	0.01	0.09
Ash content (%)	0.72	0.73	0.72	0.69	0.66	0.01	0.60
Moisture (%)	84.69	85.26	84.32	83.84	85.31	0.35	0.70
Total cholesterol (mg/g)	0.13	0.10	0.33	0.30	0.24	0.05	0.47
Triglyceride (mg/g)	1.20	0.96	0.88	0.26	0.32	0.14	0.12
Riboflavin (ug/g)	12.77	14.00	10.45	12.80	14.82	0.76	0.49
Thiamine (ug/g)	3.88 ^a^	12.08 ^b^	8.39 ^ab^	6.03 ^ab^	2.70 ^a^	1.17	0.04
Yolk							
Glucose (mg/g)	2.36	2.39	2.08	2.25	2.51	0.05	0.07
Grude protein (%)	15.02	14.27	14.34	15.04	2.51	0.18	0.23
Total soluble sugar (mg/g)	2.70 ^a^	2.86 ^ab^	3.08 ^b^	2.61 ^a^	2.71 ^a^	0.05	0.014
Crude fat (%)	29.63	29.83	29.95	29.37	31.37	0.29	0.20
Ash content (%)	1.51	1.47	1.48	1.51	1.57	0.01	0.07
Moisture (%)	52.94	53.53	53.35	53.34	51.05	0.36	0.15
Total cholesterol (mg/g)	8.70	10.59	9.84	10.25	11.39	0.44	0.42
Triglyceride (mg/g)	26.08	32.40	29.13	31.17	32.42	0.91	0.10
Riboflavin (ug/g)	11.13 ^bc^	12.31 ^c^	10.44 ^bc^	8.98 ^ab^	7.51 ^a^	0.54	0.01
Thiamine (ug/g)	3.80	6.17	4.68	5.76	2.69	0.49	0.11

^a–c^ Data within the same row marked with different small letters above the data indicate statistically significant differences (*p* < 0.05), while the same or no small letters indicate non-significant differences (*p* > 0.05).

**Table 6:** Effects of oregano oil on mineral elements of egg.

Items	Concentration (mg/kg)					SEM	*p* Value
	0	25	50	75	100		
Egg white							
Potassium (mg/kg)	0.0052	0.0050	0.0062	0.0044	0.0054	0.02	0.11
Calcium (mg/kg)	129.41	198.56	209.63	193.91	274.80	26.27	0.60
Total phosphorus (mg/g)	0.08 ^a^	0.08 ^ab^	0.11 ^b^	0.12 ^b^	0.13 ^c^	0.01	0.04
Magnesium (mg/g)	0.17	0.21	0.25	0.19	0.25	0.01	0.31
Sodium (mg/g)	1.88	2.11	2.16	1.93	2.19	0.08	0.72
Iron (mg/kg)	41.44	46.20	34.73	29.32	74.68	6.55	0.21
Selenium (ug/kg)	30.97	29.67	36.88	33.07	44.87	2.62	0.39
Zinc (mg/kg)	1.29	0.71	1.21	0.51	1.39	0.16	0.36
Yolk							
Potassium (mg/kg)	0.0030	0.0036	0.0040	0.0034	0.0039	0.01	0.19
Calcium (mg/kg)	1423.16	1553.55	2032.61	1558.43	1539.27	130.18	0.68
Total phosphorus (mg/g)	4.55	4.77	4.79	4.79	5.50	0.16	0.45
Magnesium (mg/g)	0.27	0.31	0.41	0.29	0.28	0.03	0.50
Sodium (mg/g)	0.60	0.70	0.71	0.75	0.66	0.03	0.56
Iron (mg/kg)	194.49	222.00	240.99	170.14	166.01	12.08	0.21
Selenium (ug/kg)	150.87 ^ab^	387.19 ^c^	348.77 ^c^	228.43 ^bc^	39.72 ^a^	39.26	0.00
Zinc (mg/kg)	44.51	47.54	46.12	47.79	52.15	1.69	0.74

^a–c^ Data within the same row marked with different small letters above the data indicate statistically significant differences (*p* < 0.05), while the same or no small letters indicate non-significant differences (*p* > 0.05).

## Data Availability

Data will be available upon request from the corresponding author.

## References

[1] Miranda J.M., Anton X., Redondo-Valbuena C., Roca-Saavedra P., Rodriguez J.A., Lamas A., Franco C.M., Cepeda A. (2015). Egg and Egg-Derived Foods: Effects on Human Health and Use as Functional Foods. Nutrients.

[2] Réhault-Godbert S., Guyot N., Nys Y. (2019). The Golden Egg: Nutritional Value, Bioactivities, and Emerging Benefits for Human Health. Nutrients.

[3] Lordelo M., Cid J., Cordovil C.M.D.S., Alves S.P., Bessa R.J.B., Carolino I. (2020). A Comparison between the Quality of Eggs from Indigenous Chicken Breeds and That from Commercial Layers. Poult. Sci..

[4] Vlčková J., Tůmová E., Míková K., Englmaierová M., Okrouhlá M., Chodová D. (2019). Changes in the Quality of Eggs during Storage Depending on the Housing System and the Age of Hens. Poult. Sci..

[5] Zhang J., Chen J., Yang J., Gong S., Zheng J., Xu G. (2021). Effects of Lard and Vegetable Oils Supplementation Quality and Concentration on Laying Performance, Egg Quality and Liver Antioxidant Genes Expression in Hy-Line Brown. Animals.

[6] Greenwell M., Rahman P.K.S.M. (2015). Medicinal Plants: Their Use in Anticancer Treatment. Int. J. Pharm. Sci. Res..

[7] Esmaeili Y., Paidari S., Baghbaderani S.A., Nateghi L., Al-Hassan A.A., Ariffin F. (2022). Essential Oils as Natural Antimicrobial Agents in Postharvest Treatments of Fruits and Vegetables: A Review. J. Food Meas. Charact..

[8] Leyva-López N., Gutiérrez-Grijalva E.P., Vazquez-Olivo G., Heredia J.B. (2017). Essential Oils of Oregano: Biological Activity beyond Their Antimicrobial Properties. Molecules.

[9] Al-Hijazeen M., Mendonca A., Lee E.J., Ahn D.U. (2018). Effect of Oregano Oil and Tannic Acid Combinations on the Quality and Sensory Characteristics of Cooked Chicken Meat. Poult. Sci..

[10] Asensio C.M., Paredes A.J., Martin M.P., Allemandi D.A., Nepote V., Grosso N.R. (2017). Antioxidant Stability Study of Oregano Essential Oil Microcapsules Prepared by Spray-Drying. J. Food. Sci..

[11] Gilling D.H., Kitajima M., Torrey J.R., Bright K.R. (2014). Antiviral Efficacy and Mechanisms of Action of Oregano Essential Oil and Its Primary Component Carvacrol against Murine Norovirus. J. Appl. Microbiol..

[12] Xin Y., Liu H., Yan X., Huang W., Pan S., Zhou M., Lu B., Tan B., Dong X., Yang Y. (2022). Effect of Dietary Oregano Oil on Growth Performance, Disease Resistance, Intestinal Morphology, Immunity, and Microbiota of Hybrid Grouper (*Epinephelus fuscoguttatus* ♀ × *Epinephelus lanceolatus* ♂). Front. Mar. Sci..

[13] Nadia R., Hassan R.A., Qota E.M., Fayek H.M. (2008). Effect of Natural Antioxidant on Oxidative Stability of Eggs and Productive and Reproductive Performance of Laying Hens. Int. J. Poult. Sci..

[14] Ramirez S.Y., Peñuela-Sierra L.M., Ospina M.A. (2021). Effects of Oregano (*Lippia origanoides*) Essential Oil Supplementation on the Performance, Egg Quality, and Intestinal Morphometry of Isa Brown Laying Hens. Vet. World.

[15] Gao F., Zhang L., Li H., Xia F., Bai H., Piao X., Sun Z., Cui H., Shi L. (2022). Dietary Oregano Essential Oil Supplementation Influences Production Performance and Gut Microbiota in Late-Phase Laying Hens Fed Wheat-Based Diets. Animal.

[16] Rattanawut J., Pimpa O., Yamauchi K.E. (2018). Effects of Dietary Bamboo Vinegar Supplementation on Performance, Eggshell Quality, Ileal Microflora Composition, and Intestinal Villus Morphology of Laying Hens in the Late Phase of Production. Anim. Sci. J..

[17] Li W., Xu B., Wang L., Sun Q., Deng W., Wei F., Ma H., Fu C., Wang G., Li S. (2021). Effects of Clostridium Butyricum on Growth Performance, Gut Microbiota and Intestinal Barrier Function of Broilers. Front. Microbiol..

[18] Montalto M., D’Onofrio F., Gallo A., Cazzato A., Gasbarrini G. (2009). Intestinal Microbiota and Its Functions. Dig. Liver Dis. Suppl..

[19] Wei S., Morrison M., Yu Z. (2013). Bacterial Census of Poultry Intestinal Microbiome. Poult. Sci..

[20] Pan D., Yu Z. (2014). Intestinal Microbiome of Poultry and Its Interaction with Host and Diet. Gut Microbes.

[21] (2004). Feeding Standard of Chicken.

[22] (2009). Technical Specifications for Breeding of Lhasa White Chicken (Breed Group).

[23] Applegate T.J., Angel R. (2014). Nutrient requirements of poultry publication: History and need for an update. J. Appl. Poult. Res..

[24] (2016). National Food Safety Standard—Limits of Microbial Contaminants in Foods.

[25] (2016). National Food Safety Standard—Determination of Total Dietary Fiber in Foods.

[26] (2016). National Food Safety Standard—Determination of Mycotoxins in Foods.

[27] (2016). National Food Safety Standard—Determination of Residues of Pesticides in Foods.

[28] (2016). National Food Safety Standard—Determination of Moisture in Foods.

[29] Tu K., Zhao L., Pan L.Q. (2004). Status of Examination for Egg Quality. China Poult..

[30] (2016). National Food Safety Standard—Determination of Heavy Metals in Foods.

[31] He X., Hao D., Liu C., Zhang X., Xu D., Xu X., Wang J., Wu R. (2016). Effect of Supplemental Oregano Essential Oils in Diets on Production Performance and Relatively Intestinal Parameters of Laying Hens. Am. J. Respir. Cell Mol. Biol..

[32] Ding X., Yu Y., Su Z., Zhang K. (2017). Effects of Essential Oils on Performance, Egg Quality, Nutrient Digestibility and Yolk Fatty Acid Profile in Laying Hens. Anim. Nutr..

[33] Feng J., Lu M., Wang J., Zhang H., Qiu K., Qi G., Wu S. (2021). Dietary Oregano Essential Oil Supplementation Improves Intestinal Functions and Alters Gut Microbiota in Late-Phase Laying Hens. J. Anim. Sci. Biotechnol..

[34] Grobas S., Mendez J., De Blas C., Mateos G.G. (1999). Influence of Dietary Energy, Supplemental Fat and Linoleic Acid Concentration on Performance of Laying Hens at Two Ages. Br. Poult. Sci..

[35] Chowdhury S., Mandal G.P., Patra A.K., Kumar P., Samanta I., Pradhan S., Samanta A.K. (2018). Different Essential Oils in Diets of Broiler Chickens: 2. Gut Microbes and Morphology, Immune Response, and Some Blood Profile and Antioxidant Enzymes. Anim. Feed Sci. Technol..

[36] Li F., Yin Y., Tan B., Kong X., Wu G. (2011). Leucine Nutrition in Animals and Humans: mTOR Signaling and Beyond. Amino Acids.

[37] Wu G. (2014). Dietary Requirements of Synthesizable Amino Acids by Animals: A Paradigm Shift in Protein Nutrition. J. Anim. Sci. Biotechnol..

[38] Simopoulos A.P., Salem N. (1992). Egg Yolk as a Source of Long-Chain Polyunsaturated Fatty Acids in Infant Feeding. Am. J. Clin Nutr..

[39] Shahidi F., Ambigaipalan P. (2018). Omega-3 Polyunsaturated Fatty Acids and Their Health Benefits. Annu. Rev. Food. Sci. Technol..

[40] Sokoła-Wysoczańska E., Wysoczański T., Wagner J., Czyż K., Bodkowski R., Lochyński S., Patkowska-Sokoła B. (2018). Polyunsaturated Fatty Acids and Their Potential Therapeutic Role in Cardiovascular System Disorders—A Review. Nutrients.

[41] Filipiak-Florkiewicz A., Deren K., Florkiewicz A., Topolska K., Juszczak L., Cieslik E. (2009). Quail Egg Yolk (*Coturnix coturnix* Japonica) Enriched with Omega-3 Fatty Acids. LWT-Food Sci. Technol..

[42] Bölükbasi S.C., Erhan M.K., Ürüsan H. (2010). The Effects of Supplementation of Bergamot Oil (*Citrus bergamia*) on Egg Production, Egg Quality, Fatty Acid Composition of Egg Yolk in Laying Hens. J. Poult. Sci..

[43] Abdel-Wareth A.A.A. (2016). Effect of Dietary Supplementation of Thymol, Synbiotic and Their Combination on Performance, Egg Quality and Serum Metabolic Profile of Hy-Line Brown Hens. Br. Poult. Sci..

[44] Cherian G., Quezada N. (2016). Egg Quality, Fatty Acid Composition and Immunoglobulin Y Content in Eggs from Laying Hens Fed Full Fat Camelina or Flax Seed. J. Anim. Sci. Biotechnol..

[45] Li J., Huang Q., Yang C., Yu C., Zhang Z., Chen M., Ren P., Qiu M. (2023). Molecular Regulation of Differential Lipid Molecule Accumulation in the Intramuscular Fat and Abdominal Fat of Chickens. Genes.

[46] Stephanie W., Karen A. (2018). Effect of Variety and Environment on the Amount of Thiamine and Riboflavin in Cereals and Grain Legumes. Anim. Feed Sci. Technol..

[47] Amer S.A., Tolba S.A., AlSadek D.M.M., Abdel Fattah D.M., Hassan A.M., Metwally A.E. (2021). Effect of Supplemental Glycerol Monolaurate and Oregano Essential Oil Blend on the Growth Performance, Intestinal Morphology, and Amino Acid Digestibility of Broiler Chickens. BMC Vet. Res..

[48] Abdelqader A., Al-Fataftah A.R., Daş G. (2013). Effects of Dietary Bacillus Subtilis and Inulin Supplementation on Performance, Eggshell Quality, Intestinal Morphology and Microflora Composition of Laying Hens in the Late Phase of Production. Anim. Feed Sci. Technol..

[49] Manore M.M. (2000). Effect of Physical Activity on Thiamine, Riboflavin, and Vitamin B-6 Requirements123. Am. J. Clin. Nutr..

[50] Calvo M.S., Moshfegh A.J., Tucker K.L. (2014). Assessing the Health Impact of Phosphorus in the Food Supply: Issues and Considerations123. Adv. Nutr..

[51] Chemical Composition of Eggs and Egg Products. https://link.springer.com/referenceworkentry/10.1007/978-3-642-36605-5_28.

[52] Rayman M.P. (2000). The Importance of Selenium to Human Health. Lancet.

[53] Rayman M.P. (2012). Selenium and Human Health. Lancet.

[54] Pilarczyk B., Tomza-Marciniak A., Pilarczyk R., Kuba J., Hendzel D., Udała J., Tarasewicz Z. (2019). Eggs as a Source of Selenium in the Human Diet. J. Food Compos. Anal..

[55] Oviedo-Rondón E.O. (2019). Holistic View of Intestinal Health in Poultry. Anim. Feed Sci. Technol..

[56] Apajalahti J. (2005). Comparative Gut Microflora, Metabolic Challenges, and Potential Opportunities. J. Appl. Poult. Res..

[57] Gibiino G., Lopetuso L.R., Scaldaferri F., Rizzatti G., Binda C., Gasbarrini A. (2018). Exploring Bacteroidetes: Metabolic Key Points and Immunological Tricks of Our Gut Commensals. Diges. Liver. Dis..

[58] Karlsson F.H., Ussery D.W., Nielsen J., Nookaew I. (2011). A closer look at bacteroides: Phylogenetic relationship and genomic implications of a life in the human gut. Microb. Ecol..

[59] Li Y., Xu Q., Huang Z., Lv L., Liu X., Yin C., Yan H., Yuan J. (2016). Effect of Bacillus Subtilis CGMCC 1.1086 on the Growth Performance and Intestinal Microbiota of Broilers. J. Appl. Microbiol..

[60] Sergeant M.J., Constantinidou C., Cogan T.A., Bedford M.R., Penn C.W., Pallen M.J. (2014). Extensive Microbial and Functional Diversity within the Chicken Cecal Microbiome. PLoS ONE.

[61] Wang W.W., Wang J., Zhang H.J., Wu S.J., Qi G.H. (2020). Effects of Clostridium butyricum on production performance and intestinal absorption function of laying hens in the late phase of production. Anim. Feed Sci. Technol..

[62] Shimizu J., Kubota T., Takada E., Takai K., Fujiwara N., Arimitsu N., Ueda Y., Wakisaka S., Suzuki T., Suzuki N. (2019). Relative Abundance of Megamonas Hypermegale and Butyrivibrio Species Decreased in the Intestine and Its Possible Association with the T Cell Aberration by Metabolite Alteration in Patients with Behcet’s Disease (210 Characters). Clin. Rheumatol..

